# Description of an oral Chagas disease outbreak in Venezuela, including a vertically transmitted case

**DOI:** 10.1590/0074-02760170009

**Published:** 2017-08

**Authors:** Belkisyolé Alarcón de Noya, Gladymar Pérez-Chacón, Zoraida Díaz-Bello, Sonia Dickson, Arturo Muñoz-Calderón, Carlos Hernández, Yadira Pérez, Luciano Mauriello, Eyleen Moronta

**Affiliations:** 1 Universidad Central de Venezuela, Facultad de Medicina, Instituto de Medicina Tropical, Sección de Inmunología, Caracas, Venezuela; 2 Universidad Central de Venezuela, Facultad de Medicina, Escuela de Medicina José María Vargas, Parasitología, Caracas, Venezuela; 3 Hospital de Clínicas Caracas, Departamento de Anatomía Patológica, Caracas, Venezuela; 4 Hospital de Clínicas Caracas, Departamento de Obstetricia y Ginecología, Caracas, Venezuela; 5 Clínica Sanitas Santa Paula, Caracas, Venezuela

**Keywords:** Chagas disease, oral transmission, outbreaks, vertical transmission, Venezuela

## Abstract

We describe the eleventh major outbreak of foodborne *Trypanosoma cruzi* transmission in urban Venezuela, including evidence for vertical transmission from the index case to her fetus. After confirming fetal death at 24 weeks of gestation, pregnancy interruption was performed. On direct examination of the amniotic fluid, trypomastigotes were detected. *T. cruzi* specific-polymerase chain reaction (PCR) also proved positive when examining autopsied fetal organs. Finally, microscopic fetal heart examination revealed amastigote nests. Acute orally transmitted Chagas disease can be life threatening or even fatal for pregnant women and unborn fetuses owing to vertical transmission. There is therefore an urgent need to improve national epidemiologic control measures.

Although mother-to-child transmission of *Trypanosoma cruzi* has previously been reported in Venezuela, it has been overlooked for many decades (Dao 1949, Gavaller 1953). However, vertical transmission is now beginning to again be accepted to play a role in Chagas disease (Alarcón de Noya et al. 2015). Changes in the epidemiological determinants of disease now suggest that inadvertent oral ingestion of contaminated *T. cruzi* food is the predominant infection route for expectant mothers. Infection can then be vertically transmitted to the unborn fetus (Alarcón de Noya et al. 2015). The first reports of this transmission route were described in the context of school and family outbreaks in Venezuela in 2010, including fatal outcomes for pregnant women and fetuses (Suárez et al. 2010, Alarcón de Noya et al. 2016). Subsequent evidence for vertical transmission emerged in Brazil in 2011 (Pinto et al. 2011). Up until December 2016, there have been fourteen separate outbreaks of oral Chagas disease in Venezuela, with reports of mother-to-fetus transmission having occurred during the second (Suárez et al. 2010, Alarcón de Noya et al. 2016) and eleventh outbreaks. This eleventh outbreak is the focus of our research.

In February 2015, an otherwise healthy 31-year-old woman during her twelfth week of pregnancy (index case) presented at a private clinic in Caracas with a 5-day history of fever and centripetal rash. Nine days before commencement of symptoms, the patient ate yucca and dips, and drank beer and homemade iced tea, at a family celebration in Guatire (Miranda state). A week after admission, facial edema and asthenia were noted. Simultaneously, a cousin of the index case who had shared the same food and beverages reported similar symptoms. The second patient (patient 2) was a 13-year-old female with a fever reaching 39ºC within 15 days. Symptoms included chills, facial edema, tachycardia, leukopenia, lymphopenia, thrombocytopenia, positive C-reactive protein, and increased globulin fraction. Patient 2 was given a provisional diagnosis of dengue fever on presentation.

Blood samples for diagnosis were collected from two symptomatic and six asymptomatic contacts who had shared food and beverages with the patients. An ELISA specific to the delipidised antigen of *T. cruzi* epimastigotes was performed (Maekelt 1960, González et al. 2005). All sera were also screened for specific IgG, IgA, and IgM. For demonstration of parasite infection, unstained and Giemsa-stained thin smears were assessed for the presence of trypomastigotes. Aliquots of blood (2 mL) were collected by venipuncture in sodium citrate tubes and a portion cultured in biphasic medium. These cultures were observed every 10 days over a 3-month period. Additionally, mice were inoculated intraperitoneally with 300 mL of blood and examined weekly (WHO 2002). For DNA extraction, 5 mL of blood was mixed with an equal volume of 6 M guanidine HCl /0.2 M EDTA (Schijman et al. 2003). Polymerase chain reaction (PCR) amplification was performed targeting a 188-bp fragment of *T. cruzi* nuclear DNA (Sturm et al. 1989). Electrocardiogram recordings and echocardiogram were obtained from symptomatic patients. Written informed consent was obtained from all participants or their legal guardians.

A suspected Chagas disease case was defined as any person with an epidemiological link to the family celebration that occurred in February 2015, who also went on to develop fever or other clinical manifestations. A confirmed Chagas disease case was defined as a suspected Chagas disease (including asymptomatic cases with epidemiological links) that possessed blood parasites or specific anti-*T. cruzi* antibodies with a positive PCR (WHO 2002, Alarcón de Noya et al. 2010).

*T. cruzi* DNA was detected in the peripheral blood of the index case and patient 2 by PCR (Fig. 1). Specific IgG against *T. cruzi* was also determined to be present, confirming acute Chagas disease. Specific IgM assays and conventional parasitological methods were negative, although some IgA activity against *T. cruzi* was detected in patient 2. The index case’s electrocardiogram and echocardiogram were normal and, in view of her pregnancy, benznidazole treatment was withheld. Patient 2’s echocardiogram did not show any abnormalities, but 24-hour assessment with a Holter monitor demonstrated an incomplete right bundle branch block. *T. cruzi*-specific IgM, IgG, PCR, and other conventional parasitological diagnostic methods were each negative in the six epidemiology-linked but asymptomatic cases.

**Fig. 1:**
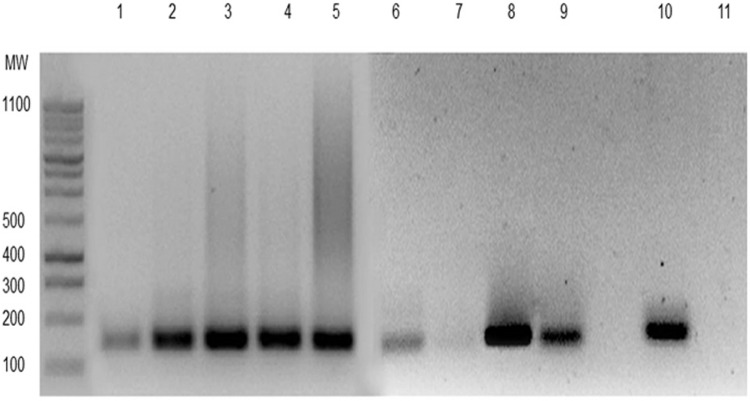
: polymerase chain reaction (PCR) to detect *Trypanosoma cruzi* DNA in the maternal index case was performed on the peripheral blood (1), placenta (2-5), fetal liver (6), fetal spleen (7), fetal kidneys (8), and the amniotic fluid (9). Samples were positive for a 188-bp amplicon corresponding to the nuclear DNA sequence of *T. cruzi.* DNA amplification of the parasite in fetal kidneys and placenta exhibited greater band intensity when compared to other organs in which *T. cruzi* DNA was also detected. Positive (10) and negative controls are also shown (11). These assays demonstrate the presence of parasites in the tissue and circulation of the index patient and the fetus.

Hydrops fetalis and fetal death were detected by ultrasound at 24 weeks of gestation in the index case. A micro-Caesarean section and fetal autopsy were performed. More than 10 mobile trypomastigotes per 100 microscopic fields (400×) were detected on direct examination of the amniotic fluid from the index case. The presence of *T. cruzi* was validated by culturing. Fetal anatomopathological macroscopic findings are described in Fig. 2. Microscopic examination of the fetal heart revealed *T. cruzi* amastigote nests within the cardiomyocyte cytoplasm and destruction of the normal micro-architecture. This included necrosis and lymphomononuclear infiltrate. Histologic signs of acute chorioamnionitis were observed, with *T. cruzi* amastigotes present in the histiocyte cytoplasm of some villi. *T. cruzi*-specific PCR was positive in the autopsied organ specimens and amniotic fluid (Fig. 1). The index case (post-Caesarean section) and patient 2 subsequently received a 60-day course of benznidazole treatment (7.5 mg/kg/day).

**Fig. 2:**
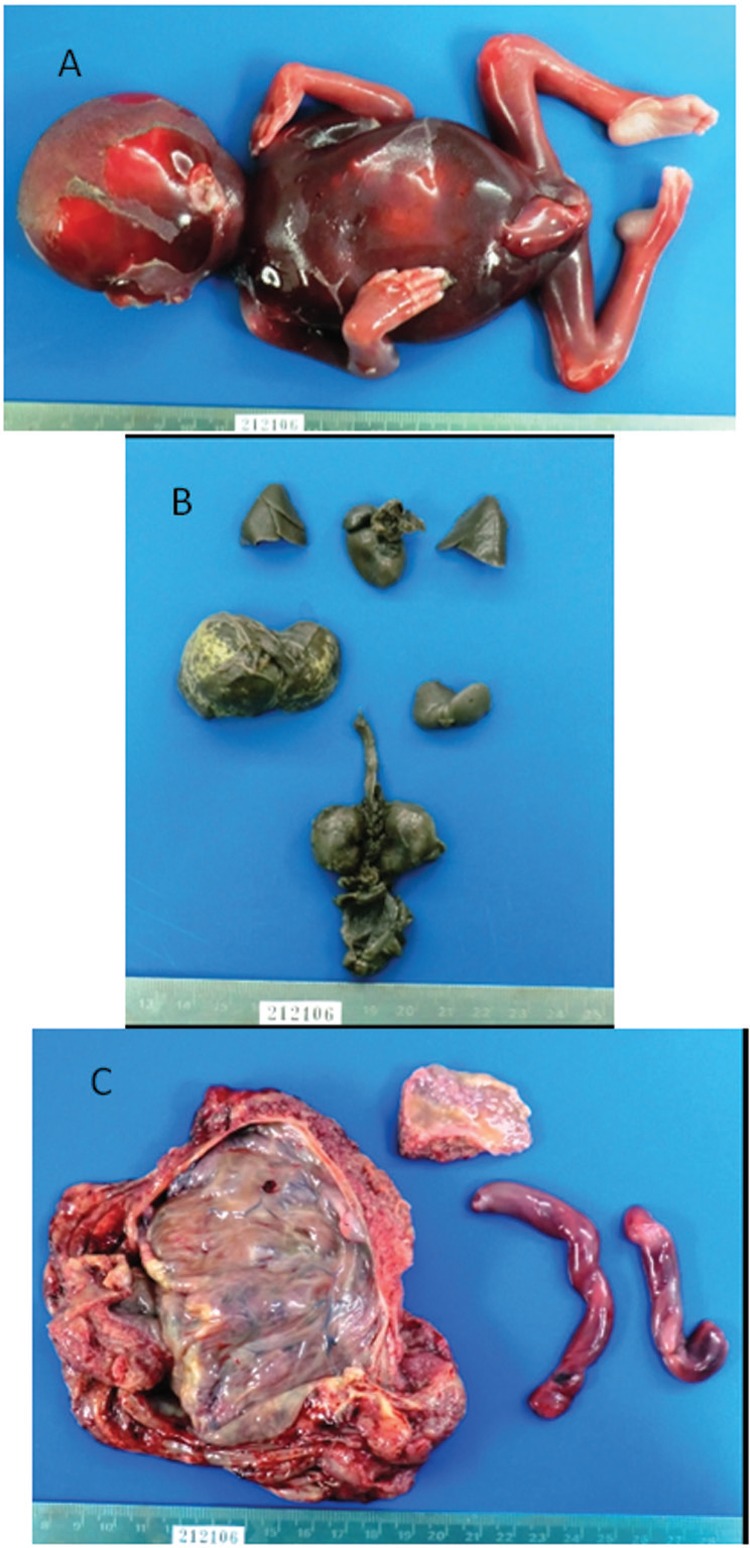
: (A) anatomopathological evaluation revealed a 320 g macerated grade II, 20 ± 3-week-stillborn male fetus; (B) dissected and formalin-fixed fetal organs. Hepatomegaly and dystrophic calcifications of the liver parenchyma were noticed; (C) placental disk, with opaque green-yellowish membranes and macroscopic signs of acute chorioamnionitis and a hypermature placenta.

The simultaneous finding of serological evidence for acute Chagas disease in two symptomatic patients exposed to handmade food and beverages in Guatire, as well as molecular confirmation of acute *T. cruzi* infection and anatomopathological features consistent with vertical transmission, are highly suggestive of an orally transmitted outbreak of Chagas disease.

A third patient, a 20-year-old female cousin of the index patient, also shared food and beverages at the same time and location. She reported the simultaneous occurrence of high-grade fever during a 17-day period, cervical lymphadenopathy, and asthenia. She was initially diagnosed with cytomegalovirus (CMV) infection based on positive qualitative serology and was contacted by us two months later. Measurements of quantitative CMV IgG titers in paired samples taken four to 12 weeks apart were not performed, nor the binding affinity of IgG antibodies assessed. *T. cruzi*-specific IgG was detected in peripheral blood samples, but PCR for *T. cruzi* DNA was negative and she was unable to be initially contacted for early assessment. Failure to confirm acute Chagas disease by molecular biology methods may be explained by her late laboratory evaluation owing to the logistic difficulties of her referral to our Chagas outpatient clinic. Two supplementary immunoassays for *T. cruzi* infection that use different techniques, or even a complete CMV workup, may have clarified this enigmatic case-scenario. False-positive reactions due to acute Chagas disease acting as an interfering infection may also explain the CMV serology results. Hence, orally transmitted *T. cruzi* infection cannot be ruled out in this patient. Therefore, by definition, patient 3 could be classified as a chronic case with suspected acute orally transmitted Chagas disease. This is owing to the fact that she and her relatives had clear simultaneous clinical manifestations and positive IgG results, albeit without demonstration of circulating parasites.

Our report highlights that, in endemic regions, high clinical suspicion for orally acquired Chagas disease is needed. This is particularly relevant for pregnant women when the risks of congenital transmission and intrauterine death are included. This is compounded by the toxicity and teratogenic effects of anti-*T. cruzi* drugs and the high risk of maternal death if treatment is not given in a timely manner (Carlier et al. 2015).

In conclusion, in Venezuela, and other South American countries where foodborne transmission of *T. cruzi* remains a neglected route of infection, shared meal and beverages as an epidemiological link between patients with fever of unknown origin should not be overlooked, especially in pregnant women or women of childbearing age. Delayed assessment of suspected cases can lead to missed opportunities for diagnosis and treatment. After the eleventh oral Chagas disease outbreak that has been the focus of our research, three further outbreaks have occurred in the states of Mérida (Añez et al. 2016), Táchira (Western Venezuela), and Miranda. There is therefore an urgent need for a national improvement in epidemiologic control measures.
